# Supplementary material for: Uncovering the Genetic and Molecular Features of Huntington’s Disease in Northern Colombia
Source: Int J Mol Sci. 2023 Nov 10;24(22):16154. doi: 10.3390/ijms242216154 (PMC10671691; doi:10.3390/ijms242216154)
Supplement: Supplementary file 1 [file ijms-24-16154-s001.zip › ijms-2564289-supplementary.pdf]

```xml
<config data_dir="/home/ubuntu/htt/scalehdinput/" forward_reference="/home/ubuntu/htt/ref/4k-HD-INTER.fasta"
reverse_reference="/home/ubuntu/htt/ref/20-TypicalReverse.fasta">
  <instance_flags demultiplex="False" quality_control="True" sequence_alignment="True" atypical_realignment="True"
genotype_prediction="True" snp_calling="True"/>
  <demultiplex_flags forward_adapter="GCGACCCTGG" forward_position="5P" reverse_adapter="GCAGCGGCTG"
reverse_position="5P" error_rate="0" min_overlap="10" min_length="" max_length=""/>
  <trim_flags trim_type="Adapter" quality_threshold="5" adapter_flag="-a"
forward_adapter="GATCGGAAGAGCACACGTCTGAACTCCAGTCAC" reverse_adapter="AGATCGGAAGAGCGTCGTGTAGGGAAAGAGTGT"
error_tolerance="0.39"/>
  <alignment_flags min_seed_length="19" band_width="100" seed_length_extension="1.5" skip_seed_with_occurrence="500"
chain_drop="0.50" seeded_chain_drop="0" seq_match_score="1" mismatch_penalty="4" indel_penalty="6,6"
gap_extend_penalty="6,6" prime_clipping_penalty="5,5" unpaired_pairing_penalty="17"/>
  <prediction_flags snp_observation_threshold="2" quality_cutoff="0"/>
</config>
```

Figure S1. Configuration file used to run ScaleHD on our sequence data. This file is freely available from https://t.ly/hi2aN.

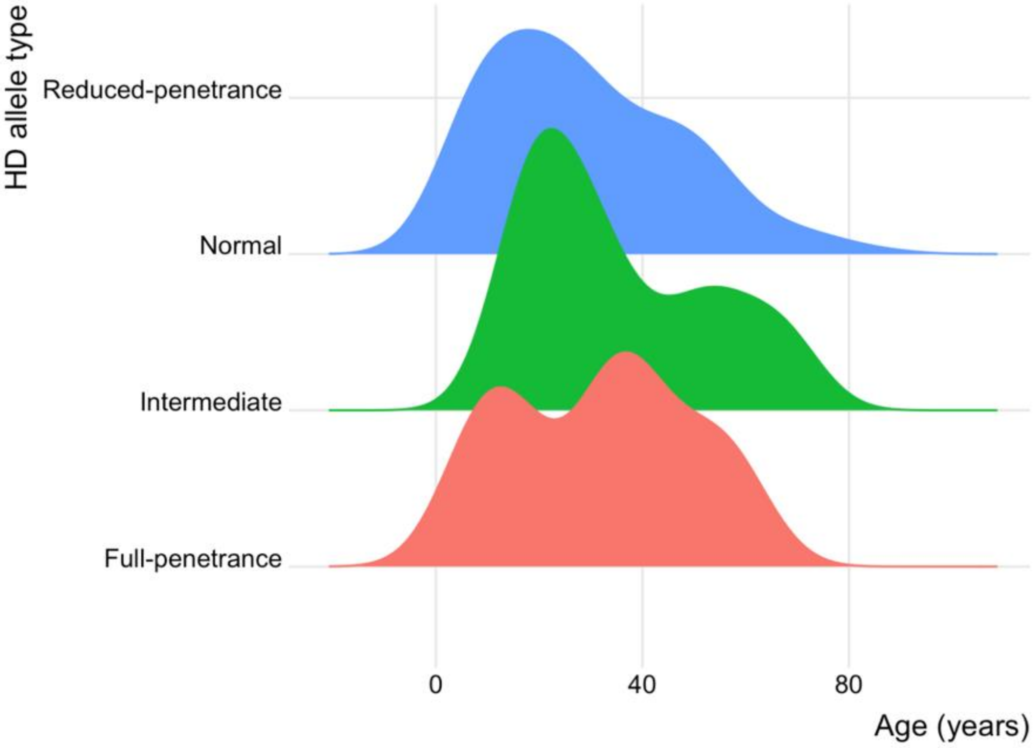

Figure S2. Age distribution by *HTT* allele type.

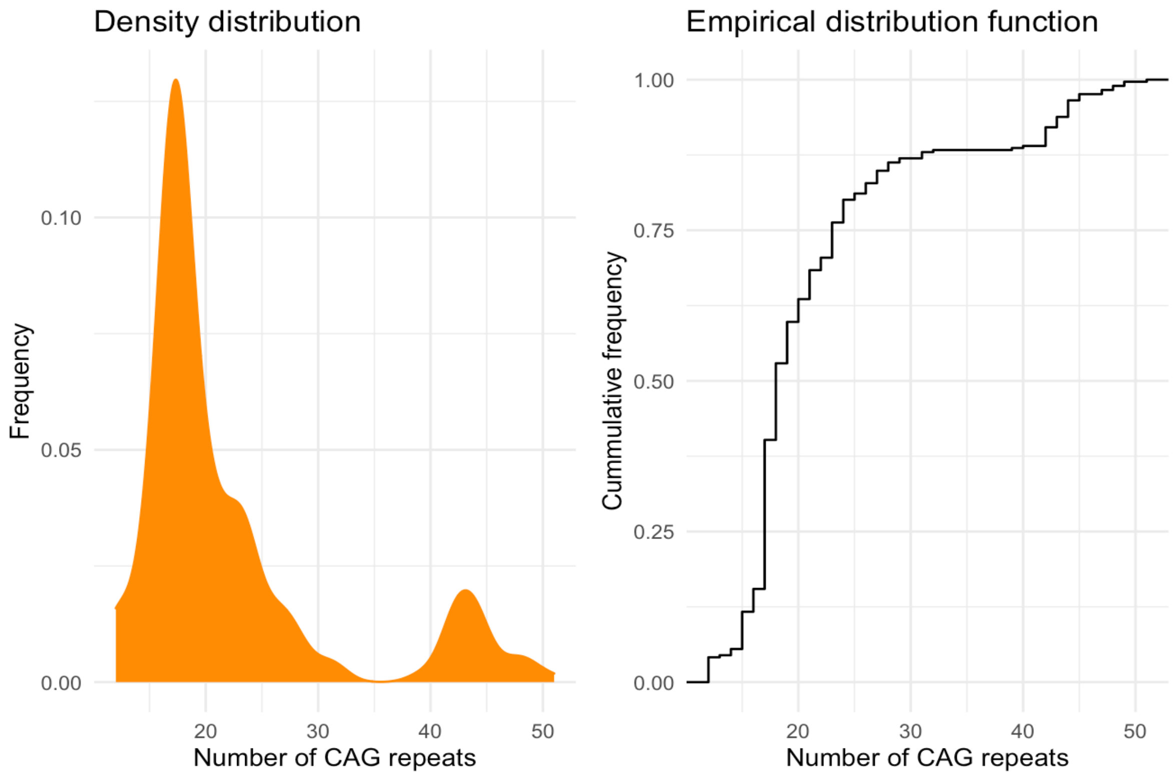

Figure S3. Distribution of *HTT* CAG repeats in a sample of 291 individuals from Juan De Acosta, Atlántico.

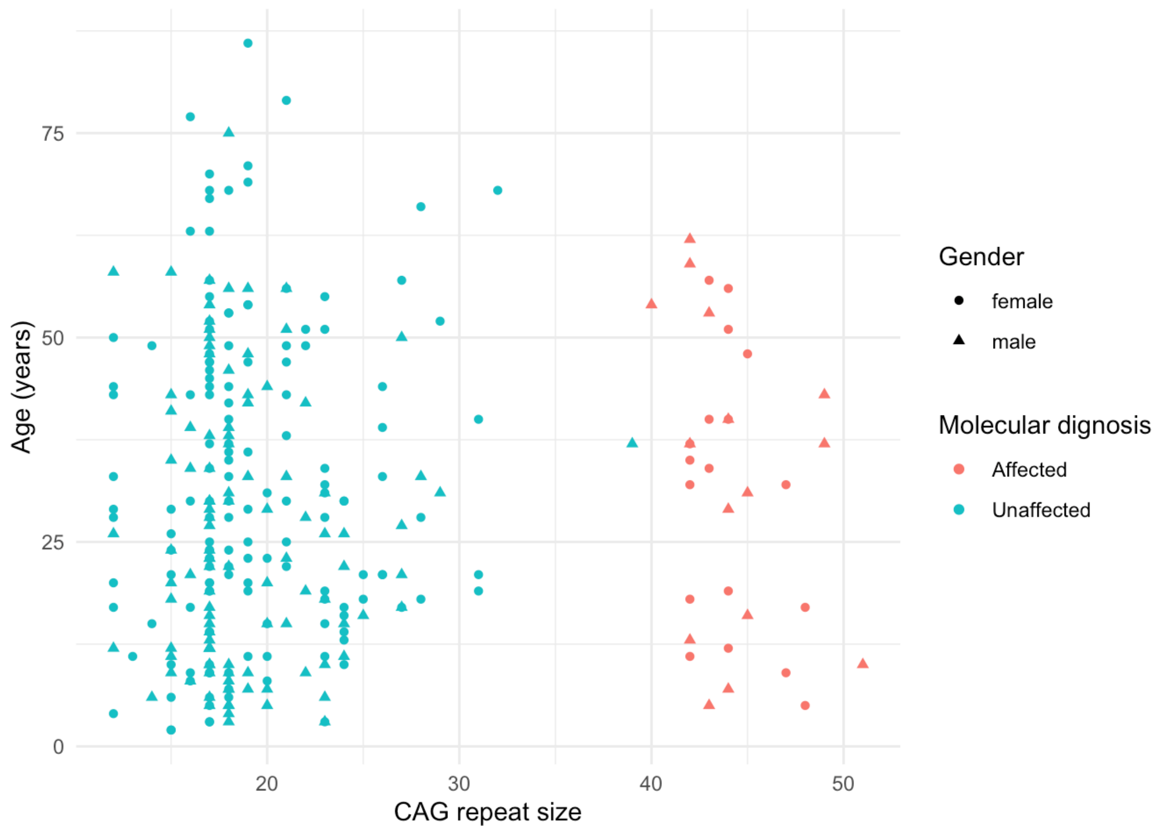

Figure S4. Age vs. Number of *HTT* CAG repeats by Molecular diagnosis (Affected: >40 CAG repeats; Not affected: ≤40 CAG repeats) in 291 individuals from Juan De Acosta, Atlántico.

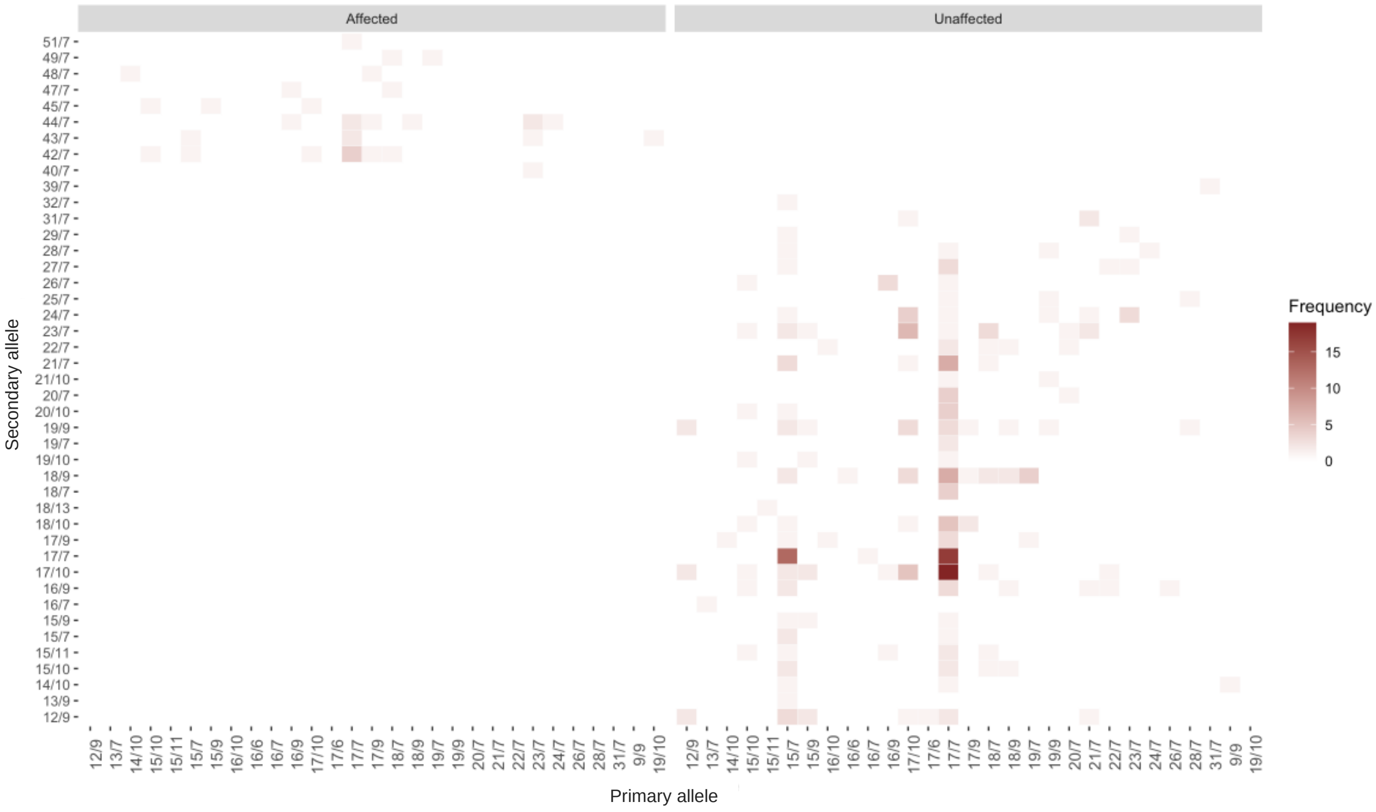

Figure S5. Allele combinations (genotypes) by Molecular diagnosis in 291 individuals from Juan De Acosta, Atlántico.

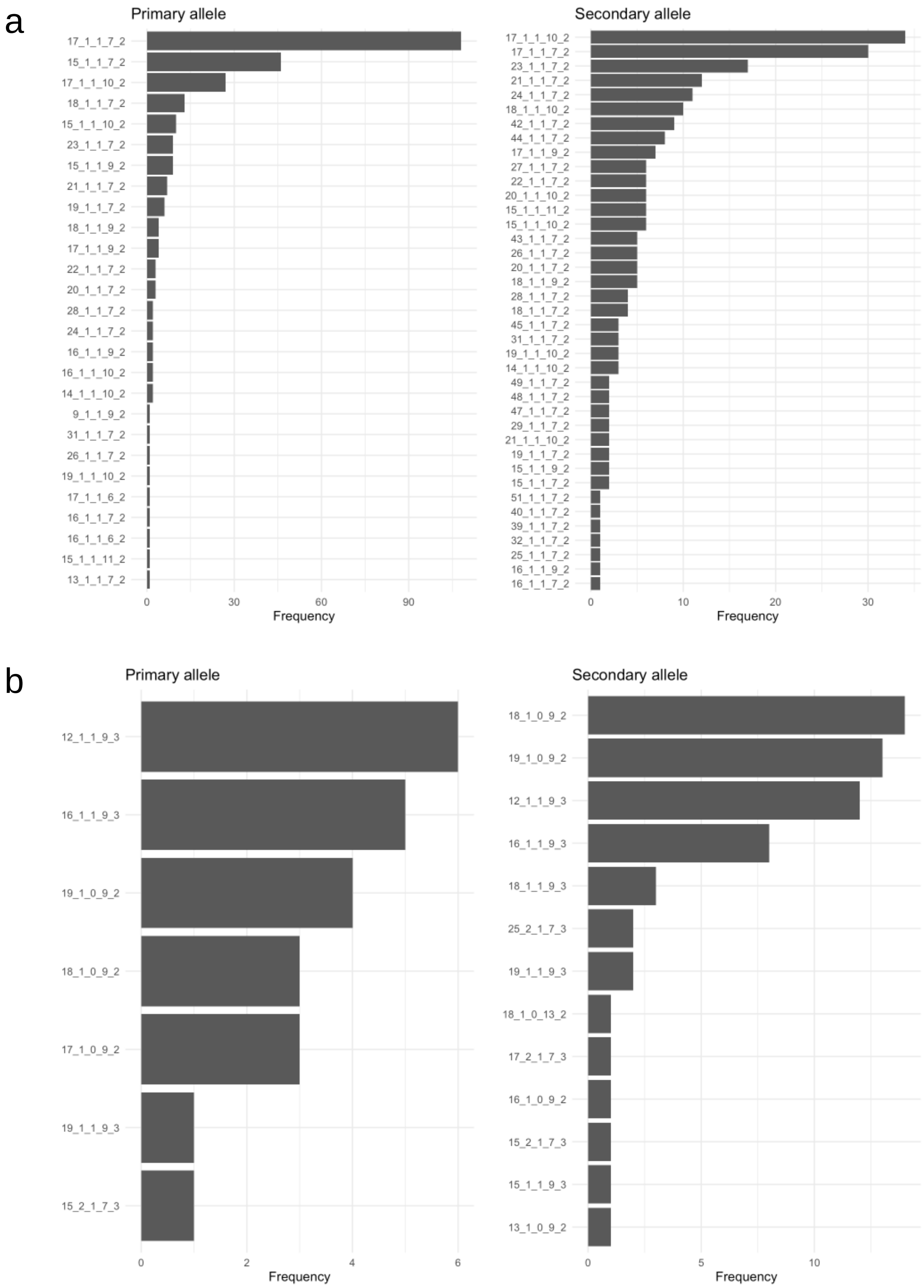

Figure S6. Frequency of **(a)** typical and **(b)** atypical sequence structures by allele in our cohort of 291 individuals. For more information, see https://scalehd.readthedocs.io/en/latest/AutomatedGenotyping.html.
